# Does nail size or difference between canal and nail diameter influence likelihood of union or time to union of femoral shaft fractures treated with intramedullary nailing? A retrospective cohort study

**DOI:** 10.1186/s12891-022-05781-0

**Published:** 2022-08-31

**Authors:** Chiu-Yu Shih, Chew-Teng Kor, Cheng-Pu Hsieh, Chiu-Liang Chen, Yu-Cheng Lo

**Affiliations:** 1 Department of Orthopedic Surgery, Changhua Christian Hospital, Changhua, 500 Taiwan; 2 Big Data Center, Changhua Christian Hospital, Changhua, 500 Taiwan; 3 Graduate Institute of Statistics and Information Science, National Changhua University of Education, Changhua, 500 Taiwan; 4 Department of Nursing, Hungkuang University, Taichung City, Taiwan

**Keywords:** Femoral fracture, Fracture Fixation, Intramedullary, Nailing, Intramedullary, Fractures, Ununited, Fractures, Malunited

## Abstract

**Background:**

This study aims to determine whether nail size or the difference between canal and nail diameter (CN difference) affects the union rate and time of femoral shaft fracture treated with an interlocking intramedullary nail (IMN).

**Methods:**

This was a retrospective review of 257 patients with femoral shaft fractures treated with IMN at a tertiary trauma medical center. All the IMN inserted were the same (Stryker T2 Femoral Nail). The patients were divided into groups based on nail size (10-, 11-, 12-, or 13-mm) and CN difference (< 1, 1–2, or > 2 mm), and union rate and time to union were compared.

**Results:**

The 10-, 11-, 12-, and 13-mm groups based on nail size had 113, 74, 54, and 16 patients, respectively. The overall union rate was 97% (257/265). No significant differences in union rate or time to union were observed among these 4 groups. The groups based on CN differences of < 1-, 1 to 2, and > 2 mm comprised 143, 79, and 35 patients, respectively. Again, no significant differences were noted in union rate or mean time to union among the groups.

**Conclusions:**

Similar union rate and time to union were observed, regardless of nail size or CN difference. This finding indicates that most simple femoral shaft fractures can be treated with a standard, reamed 10-mm IMN. A larger nail insertion is unnecessary and presents more risks; comparatively, the use of a small nail with less reaming is simpler, requires shorter operative times, results in less blood loss, and is less expensive.

## Background

Reamed intramedullary nail (IMN) is considered the standard treatment for femoral shaft fracture because it has a high union rate and low complication rate [1, 2]. Early rehabilitation with weight bearing as tolerated accelerates the recovery process. Inserting a large nail is traditionally advised because full contact between the medullary canal and nail provides stability and offers maximal torsional, bending, and axial load resistance. However, the development of metallurgy and advancement of nail design have led to the use of smaller nails as an alternative that does not compromise in strength and reduce the total amount of reaming [3]. Two recent studies have suggested that the nail size and difference between the femoral medullary canal diameter at the isthmus and intramedullary nail diameter (CN difference) do not affect the likelihood of union rate or time to union [4, 5]. However, those studies did not mention the specific IMN inserted which impacted fracture healing. IMN with sulcus around the outer surface increases contact and friction with the intramedullary canal theoretically strengthens construct stability. Furthermore, different configuration and number of screws locked within the proximal IMN affect stability. Common options include cephalomedullary fixation with a lag screw, two 5.0 mm screws with reconstruction type or obliquely toward calcar. With uniform of the nail and screws selection, our retrospective study was designed to corroborate these results with more concurrent criteria.

We investigated the relationships between IMN size and CN diameter and union time in patients with simple femoral shaft fractures. We speculated that both CN difference and nail diameter would have no significant effect on union time.

## Methods

**Fig. 1:**
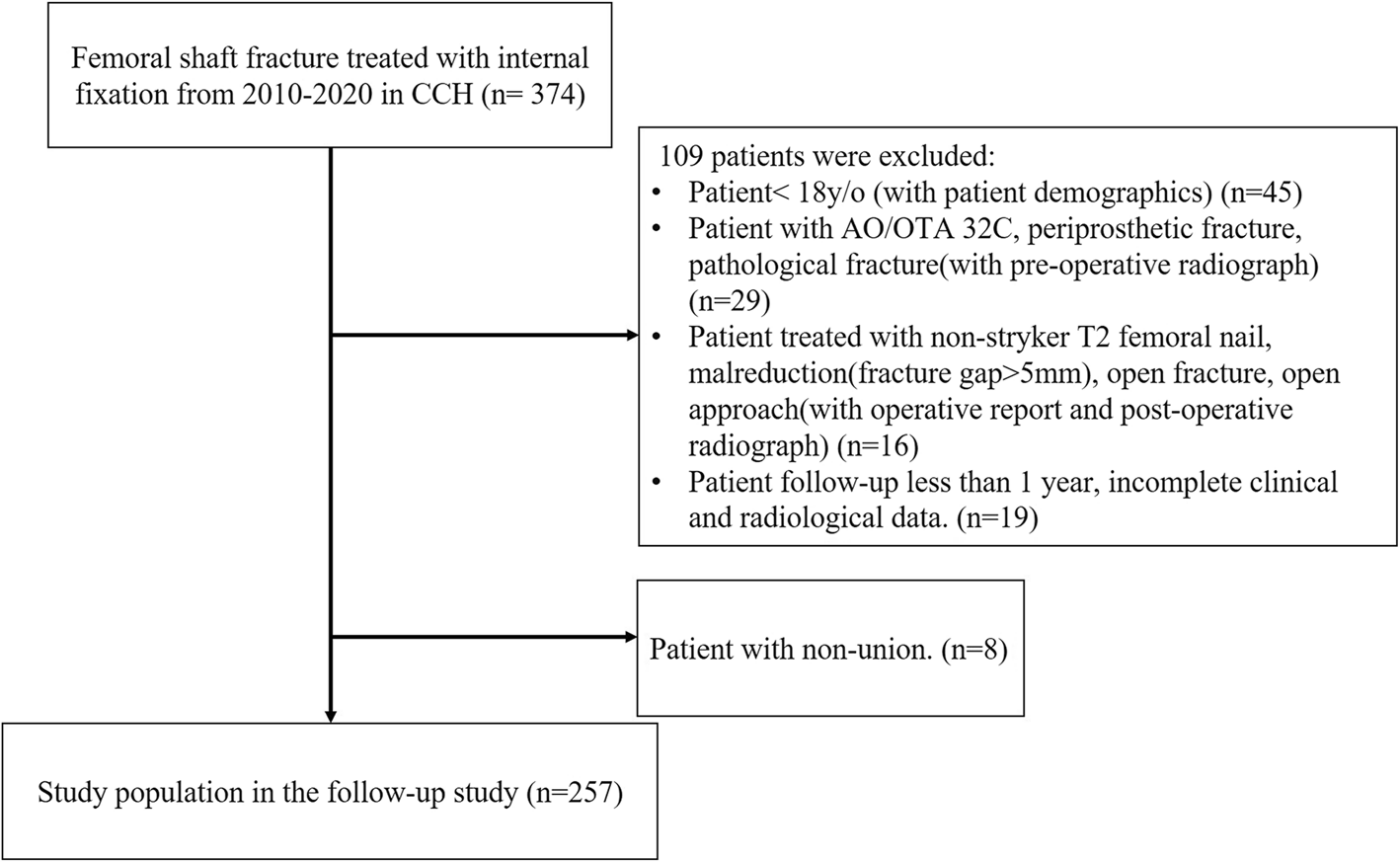
Recruitment flow chart

We conducted a retrospective case–control study of patients with diaphyseal femoral fracture at the tertiary trauma center of Changhua Christian Hospital (CCH) from 2010 to 2020. A femoral shaft fracture was defined as the fracture site located 5 cm below the lesser tuberosity and within 6 cm of the distal physeal scar. Patients with transverse, spiral, oblique, or wedge-shaped fracture patterns corresponding to AO Foundation/Orthopaedic Trauma Association (AO/OTA) classification 32A or 32B were included. Patients with segmental or fragmentary segmental fracture patterns correspond to AO/OTA 32C were excluded. All the fractures were treated with the Stryker T2 Femoral Nailing System through antegrade or retrograde insertion. The exclusion criteria were age of < 18 years, periprosthetic fracture, pathological fracture, open fracture, follow-up less than 1 year and incomplete clinical or radiological data. Patient with malreduction, which was defined as fracture gap > 5 mm at post-operative radiograph, were also excluded. All the fractures were reduced closely by traction from fracture table and manual manipulation. Sequential reaming started at 8.5 mm and ended at 1.5 or 2 mm larger than the planned nail diameter for insertion. Nail size was determined from cortical chatter and intraoperative fluoroscopic images. Three screws were locked with nail, one 5.0 mm oblique screw from the greater trochanter toward the calcar and two 5.0 mm distal screws from lateral to medial in direction. Assisted weight bearing as tolerated was the postoperative rehabilitation protocol. Age, gender, body mass index (BMI), smoking status, alcohol habit, diabetes, fracture pattern, nail size (obtained from the operative record and the product sticker from the chart), and canal diameter were recorded. Clinically, fracture union was defined as no pain on palpation of the fracture site, improved ambulation, and radiographic bridging callus formation with a minimum continuity of 3 cortices on anteroposterior and lateral images. Nonunion was defined as a clinically and radiographically ununited fracture that required further intervention, such as dynamization, exchange nailing or plate augmentation. The radiographic features indicating nonunion include a persistent fracture line at 9 months and no bridging callus formation in a time span of 3 months. In total, 265 patients qualified to our study. 8 patients revealed nonunion. The recruitment flow chart is shown in Fig. 1.

**Fig. 2:**
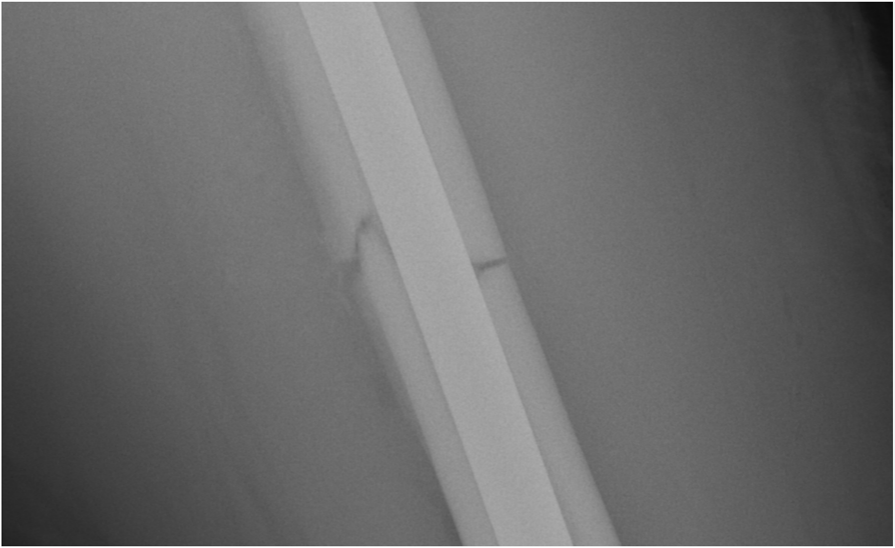
Radiographic example for CN difference < 1 mm

**Fig. 3:**
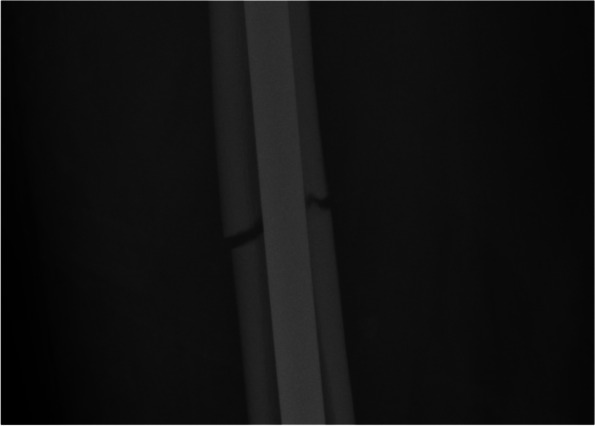
Radiographic example for CN difference 1 ~ 2 mm

**Fig. 4:**
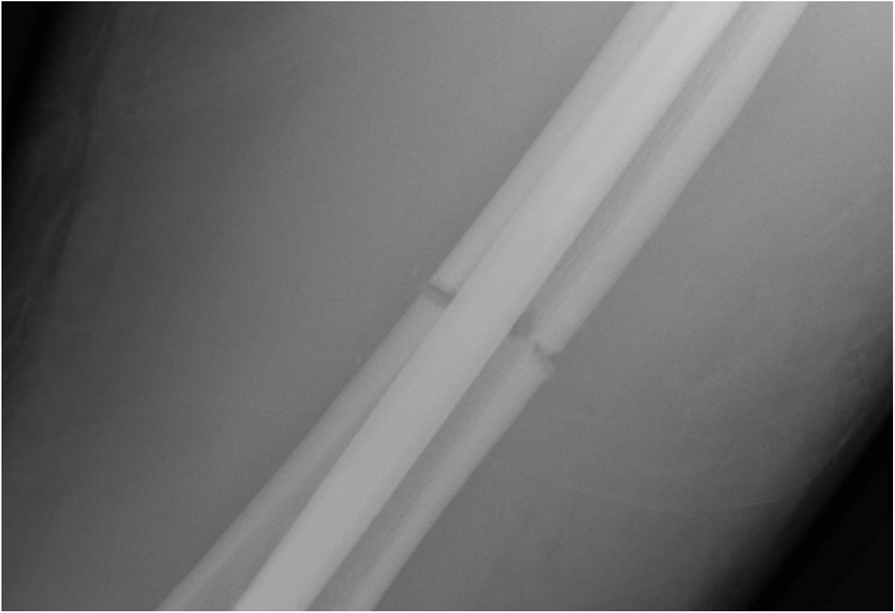
Radiographic example for CN difference > 2 mm

The remained 257 patients were divided into 4 groups based on IMN diameter: 10, 11, 12, or 13 mm. In addition, postoperative radiographs from the day of the operation were used to measure the intramedullary canal diameter at the isthmus. The digital ruler of the Picture Archiving and Communication System (PACS) was used for measurement manually by two independent trauma-trained orthopedics surgeons. We measured the canal and the nail diameter on the radiographs and calculated the canal diameter as per the size ratio of the canal to the nail. The nail diameter was obtained from the operation report. The CN difference was recorded. If there was a disagreement between the two surgeons, another trauma-trained orthopedics surgeon who was completely blinded to the measurement result would measure that case again. The final result was depended on the majority.. The patients were divided into 3 groups based on whether this difference was < 1 (Group 1), 1 to 2 (Group 2), or > 2 mm (Group 3), radiographic examples are shown in Figs. 2, 3, 4. The time to fracture union was recorded for each patient who achieved union.

This study was exempted from a full ethical review and was approved by the Changhua Christian Hospital Institutional Review Board, approval number 210626. The Changhua Christian Hospital Institution Review Board waived the requirement of informed consent based on Taiwan’s Human Subjects Research Act.

### Statistical analysis

For categorical and continuous variables, data are presented as numbers (percentages) or means and standard deviations. To compare 2 or more groups, chi-square tests were performed for categorical variables, and Student t tests or analyses of variance were performed for continuous variables. Linear regression models were used to assess the associations between patient characteristics and time to union. A scatter plot was used to visualize the association between nail size and time to union. All statistical analyses were performed using SPSS 22.0, and a 2-tailed *P* value of < 0.05 was defined as statistically significant.

## Results

### Comparison of patient clinical characteristics and outcomes related to nail diameter

The 10-mm group had 113 patients. The 11-, 12-, and 13-mm groups had 74, 65, and 16 patients, respectively. No statistical differences were observed among the groups in age, insertion method, CN difference, fracture classification distribution, smoking habits, or alcohol consumption habits (Table 1). The overall union rate was 96.8%. Notably, male patients tended to receive larger nails. A statistically significant difference was noted among groups in diabetes prevalence, which was higher in the 13-mm group than it was in the other groups. No significant difference was noted in the nonunion rate or mean time to union among the 4 groups.

**Table 1:** Demographics and fracture characteristics by nail size

	Nail size				*P* value
	10 mm	11 mm	12 mm	13 mm	
Sample Size	113	74	54	16	
Age	32.7 ± 15.8	34 ± 17.7	32.1 ± 15.8	42 ± 23.7	0.195
Age > 50	21(18.6%)	14(18.9%)	7(13%)	4(25%)	0.675
Gender					
F	55(48.7%)	24(32.4%)	12(22.2%)	4(25%)	0.004
M	58(51.3%)	50(67.6%)	42(77.8%)	12(75%)	
A/R					
A	103(91.2%)	71(95.9%)	53(98.1%)	15(93.8%)	0.277
R	10(8.8%)	3(4.1%)	1(1.9%)	1(6.3%)	
CN difference					
< 1	59(52.2%)	39(52.7%)	37(68.5%)	8(50%)	0.201
1 ~ 2	38(33.6%)	26(35.1%)	11(20.4%)	4(25%)	0.247
> 2	16(14.2%)	9(12.2%)	6(11.1%)	4(25%)	0.527
AO/OTA					
32A	87(77%)	60(81.1%)	45(83.3%)	14(87.5%)	0.651
32B	26(23%)	14(18.9%)	9(16.7%)	2(12.5%)	
BMI	24.2 ± 10.3	24.3 ± 5.8	23.8 ± 4.6	27.1 ± 6.5	0.537
BMI > 30	9(8%)	12(16.2%)	6(11.1%)	4(25%)	0.135
Smoking	22(19.5%)	17(23%)	20(37%)	5(31.3%)	0.089
Alcohol	20(17.7%)	15(20.3%)	15(27.8%)	4(25%)	0.491
DM	5(4.4%)	2(2.7%)	1(1.9%)	4(25%)	0.001
Outcome					
Union time	5.5 ± 2.2	6.1 ± 2.4	5.6 ± 2	6.3 ± 1.9	0.233
Nonunion	4(3.5%)	3(4.1%)	0(0.0%)	1(6.3%)	0.468

*F* Female, *M* Male, *A* antegrade, *R* Retrograde, *CN* Canal to Nail, *AO/OTA* AO Foundation/Orthopaedic Trauma Association classification, *BMI* Body Mass Index, *DM* Diabetes Mellitus

### Comparison of clinical characteristics and outcomes by CN difference

Groups 1, 2, and 3 consisted of 143, 79, and 35 patients, respectively. No significant differences were observed in age, gender, insertion method, nail size, BMI, smoking habits, or alcohol consumption habits. Group 2 and group 3 had higher rates of the more complex fracture pattern, 32B. Despite varied CN differences, no significant differences among the groups were noted in the nonunion rates or mean time to union (Table 2).

**Table 2:** Demographics and fracture characteristics by CN difference

	CN difference			*P* value
	Group 1 < 1 mm	Group 2 1-2 mm	Group 3 > 2 mm	
Sample size	143	79	35	
Age	32.9 ± 16.1	35.1 ± 18.8	32.4 ± 16.4	0.614
Age > 50	24(16.8%)	17(21.5%)	5(14.3%)	0.566
Gender				
F	54(37.8%)	34(43%)	7(20%)	0.060
M	89(62.2%)	45(57%)	28(80%)	
A/R				
A	135(94.4%)	75(94.9%)	32(91.4%)	0.749
R	8(5.6%)	4(5.1%)	3(8.6%)	
Nail size				
10 mm	59(41.3%)	38(48.1%)	16(45.7%)	0.601
11 mm	39(27.3%)	26(32.9%)	9(25.7%)	0.614
12 mm	37(25.9%)	11(13.9%)	6(17.1%)	0.093
13 mm	8(5.6%)	4(5.1%)	4(11.4%)	0.404
AO/OTA 32				
32A	128(89.5%)	55(69.6%)	23(65.7%)	< 0.001
32B	15(10.5%)	24(30.4%)	12(34.3%)	
BMI	24 ± 5.3	25.3 ± 12.3	23.6 ± 3.6	0.443
BMI > 30	19(13.3%)	11(13.9%)	1(2.9%)	0.196
Smoking	39(27.3%)	17(21.5%)	8(22.9%)	0.609
Alcohol	32(22.4%)	15(19%)	7(20%)	0.828
DM	7(4.9%)	3(3.8%)	2(5.7%)	0.888
Union time	5.7 ± 2.1	5.8 ± 2.3	5.7 ± 2.3	0.889
Nonunion	3(2.1%)	5(6.3%)	0(0%)	0.115

### Comparison of patient clinical characteristics and outcomes by nail insertion direction: antegrade versus retrograde

Of the 257 femoral shaft fractures, 242 were treated with antegrade nail insertion, and 15 were treated with retrograde insertion. No significant differences were noted in age, gender, CN difference, IMN size, fracture pattern, smoking habits, diabetes prevalence, or alcohol consumption habits between the patients receiving antegrade and retrograde IMN. The retrograde group had a significantly higher mean BMI than the antegrade group. Nonunion was experienced by 6 patients in the antegrade group and 2 patients in the retrograde group. The average healing time was 5.7 months in the antegrade group and 6.7 months in the retrograde group, but this difference was not statistically significant (Table 3).

**Table 3:** Demographics and fracture characteristics of patients receiving antegrade or retrograde IMN

	A/R		*P* value
	A	R	
Sample Size	242	15	
Age	32.6 ± 16.4	47.6 ± 20.9	0.016
Age > 50	39(16.1%)	7(46.7%)	0.003
Gender			
F	89(36.8%)	6(40%)	0.802
M	153(63.2%)	9(60%)	
CN difference			
< 1 mm	135(55.8%)	8(53.3%)	1.000
1 ~ 2 mm	75(31%)	4(26.7%)	1.000
> 2 mm	32(13.2%)	3(20%)	0.438
Nail size			
10 mm	103(42.6%)	10(66.7%)	0.119
11 mm	71(29.3%)	3(20%)	0.565
12 mm	53(21.9%)	1(6.7%)	0.206
13 mm	15(6.2%)	1(6.7%)	1.000
AO/OTA 32			
32A	196(81%)	10(66.7%)	0.177
32B	46(19%)	5(33.3%)	
BMI	24 ± .9	29.7 ± 7.9	0.007
BMI > 30	25(10.3%)	6(40%)	0.001
smoking	61(25.2%)	3(20%)	0.651
alcohol	51(21.1%)	3(20%)	0.921
DM	11(4.5%)	1(6.7%)	0.706
Union time	5.7 ± 2.2	6.7 ± 2.7	0.101
nonunion	6(2.5%)	2(13.3%)	0.019

### Associations between patient characteristics and time to union

A multivariate linear regression analysis showed no significant association between IMN diameter or CN difference and fracture healing. In addition, alcohol consumption, diabetes, age, and BMI showed no influence on the union time. Moreover, patients with AO/OTA 32B fracture and smoking were more likely to have a prolonged union time. The Figure visualizes the association between nail size and time to union; no linear trend is observable (Table 4).

**Table 4:** Associations between patient characteristics and time to union in linear regression for patients with successful fracture union

Variables	coefficient	Standard Error	Correlation	*P* value
Male	0.296	0.310	0.065	0.341
A/R				
A	ref	–	–	–
R	1.229	0.645	0.125	0.058
Nail size per 1 mm increase	0.203	0.153	0.087	0.186
CN difference	-0.090	0.198	-0.030	0.650
**AO/OTA 32**				
**32B vs 32A**	**0.843**	**0.364**	**0.153**	**0.021**
**Smoker vs no smoker**	**0.815**	**0.411**	**0.161**	**0.049**
Alcohol vs non-alcohol	-0.734	0.426	-0.137	0.086
DM	-0.627	0.690	-0.061	0.365
Age > 50	-0.312	0.398	-0.055	0.433
BMI > 30	-0.555	0.457	-0.081	0.225

## Discussion

This study was designed to corroborate the results of previous studies suggesting that fracture healing is unrelated to IMN diameter and CN difference in patients with femoral shaft fractures treated with IMN. High union rate was observed for simple fracture patterns (AO/OTA 32A and 32B) treated with interlocking IMN. We selected patients with simple fracture in order to minimize the confounding, non-modifiable factor which may influence union rate and time to union with various degree of complexity in AO/OTA 32C fracture pattern. According to our study results, union time was unrelated to IMN size and CN difference. Multivariate linear regression revealed that, rather than either of these variables, fracture pattern and smoking habits affected time to union.

A larger IMN diameter was previously believed to be more effective at providing adequate stability and promoting healing in the load-sharing device. Press-fit contact between the nail and medullary wall can help to minimize movement of the nail and canal to maintain reduction [6]. Therefore, inserting large-diameter nails is standard care. Using small-diameter nails may increase interfragmentary motion, which creates an unfavorable environment for union. As the load transferred through the nail increase, protracted union time、nonunion and even implant failure becomes increasingly likely. In a biomechanical test, a 12-mm IMN exhibited high endurance. Brumback et al. recommended 12-mm nails [7] and Clatworthy et al. preferred 13-mm nails for men and 12-mm nails for women [8]. Arazi et al. used 12-, 13-, and 14-mm nails in their study, which revealed optimal outcomes [9]. However, these studies were conducted over 2 decades ago. Because of improvements in nail design and metallurgy, newer nails can withstand greater compressive, torsional, and bending loads and thus enable smaller nails to achieve comparable strength to that of older, larger nails [3]. Current guidelines suggest minimal reaming after the occurrence of isthmic cortical chatter (0.5–1 mm). The appropriate nail diameter for a proper fit is 1 to 1.5 mm smaller than that of the largest reamer. The intraoperative midportion and narrowest medullary diameter can also be referenced for nail diameter selection [10].

In this retrospective study, we aimed to provide further evidence that IMN diameter and CN difference do not affect the likelihood of union or time to union. We first compared groups of patients receiving different IMN sizes and found no significant differences in union rate and mean time to union. We then compared groups with different CN differences at the isthmus. No significant difference in union rate and time to union were observed among three groups. No patients in Group 3 (> 2-mm CN difference) experienced nonunion. Patients in Group 2 and Group 3 who were treated without tight contact between the canal and nail tended to have the more complex fracture pattern in AO/OTA 32B. A complex fracture pattern may prevent physicians from inserting a tight-fitting nail. However, no significant differences were noted in union rate or time to union among the groups. Our result suggests that treating all diaphyseal femoral fractures with simple fracture patterns without tight contact between the nail and canal is reasonable; a 10-mm nail should be suitable in most cases.

Unlike patients in previous studies, all patients in our study received the same implant administered with the T2 Femoral Nailing System. This system adopts the piriformis fossa as the starting point, with one proximal and 2 distal interlocking screws, which are all 5.0 mm in size. Because of this uniformity in implants, the strength and design of the nails were consistent.

In our opinion, reduction is the most important surgeon-controlled factor affecting fracture union. In our study, patients with fracture gap > 5 mm postoperative were excluded. Furthermore, due to the nature of mid-shaft femoral fracture, all patients achieved proper sagittal and coronal plane alignment with IMN insertion in our study group. However, rotation was difficult to assess by plain film alone. Although mal-rotation resulted in cosmetic concern, shift of weight bearing axis and patella-femoral joint problem. The relationship between mal-rotation and fracture healing is not clear yet [11]. According to Millar et al., poor fracture reduction is associated with 11.5-fold greater odds of nonunion. Although this study emphasizes the importance of maximizing nail fit at the isthmus to decrease the risk of fracture non-union [12], we consider that poor nail fit is attributed to inadequate fracture reduction, which renders inserting nail appropriately impossible. The resultant non-union is actually associated with inadequate fracture reduction rather than smaller nail size. Our result further confirmed that nail size is not as important as fracture reduction.

Although reamed IMN is considered the gold standard treatment for femoral shaft fracture because of its high union rate [13]. Inserting smaller nails with limited reaming has some benefits. Limiting the reaming process minimizes alterations of the bony architecture, providing an ideal situation for osteoinduction [14]. Reducing thermal necrosis in the cortical bone to preserve blood flow. Providing surgeons with numerous options for nail exchange during revision. We avoided excessive reaming, which can elevate intramedullary pressure and lead to marrow debris leakage into the venous system. Fat embolism syndrome, acute respiratory distress syndrome, and even sudden death can occur though some studies have questioned the applicability [15, 16]. Excessive reaming also causes increase operative time and blood loss, reduce bone strength, and lead to cortical thinning [17]. In addition, a large nail can cause iatrogenic fracture propagation, iatrogenic bursting of the femoral canal, insertion difficulties. Finally, the lower cost of smaller nails is another benefit. Similar to a previous study, our study revealed no difference in union rate and time between antegrade and retrograde femoral nailing [18].

Nonetheless, our study has shortcomings. Primarily, manual measurement of the canal diameter with post-operative radiograph comes with inevitable error and is not strictly precise. In addition, malrotation, which is important in terms of reduction quality in lower limb fracture was not evaluated due to assessment difficulty with post-operative radiographic films. Furthermore, patients with CN difference > 2 mm represent only a small proportion in our study group due to traditional doctrine leading us to insert an IMN as large as possible. Finally, patients may require different rehabilitation protocols because of their concomitant health status.

## Conclusions

In our retrospective cohort study, union rate and time to union were unaffected by nail size and CN difference in simple femoral shaft fractures treated with IMN. This finding indicates that a 10-mm interlocking nail is a reasonable option in most circumstances. The use of this standard nail could avoid complications associated with the insertion of larger nails, excessive reaming and reduce cost. Larger nails could be reserved for use in revision.

## Data Availability

The dataset supporting the conclusions of this study is available upon reasonable request by contacting the corresponding author. But the primary data were not shared because of patient privacy and other studies related to these primary data were underway confidentially.

## Abbreviations

IMN: Intramedullary nail
CN difference: Difference between the diameter of Intramedullary Canal and Intramedullary Nail
F: Female
M: Male
A: Antegrade
R: Retrograde
CN: Canal to Nail
AO/OTA: AO Foundation/Orthopaedic Trauma Association
BMI: Body Mass Index
DM: Diabetes Mellitus
